# Modulation of Properties by Ion Changing Based on Luminescent Ionic Salts Consisting of Spirobi(boron ketoiminate)

**DOI:** 10.3390/molecules27113438

**Published:** 2022-05-26

**Authors:** Kazumasa Suenaga, Shunichiro Ito, Kazuo Tanaka, Yoshiki Chujo

**Affiliations:** Department of Polymer Chemistry, Graduate School of Engineering, Kyoto University, Katsura, Nishikyo-ku, Kyoto 615-8510, Japan; artificial.tai@gmail.com (K.S.); ito@poly.synchem.kyoto-u.ac.jp (S.I.); chujo@poly.synchem.kyoto-u.ac.jp (Y.C.)

**Keywords:** boron, spiro, ionic salt, luminescence

## Abstract

We report development of luminescent ionic salts consisting of the boron ketoiminate structure, which is one of the robust skeletons for expressing aggregation-induced emission (AIE) properties. From the formation of the boron-centered spiro structure with the ketoiminate ligands, we obtained stable ionic salts with variable anions. Since the ionic salts show *T*_m_s below 100 °C, it was shown that these salts can be classified as an ionic liquid. By using PF_6_ anion, the single crystal—which is applicable for X-ray crystallography—was obtained. According to the optical measurements, it was proposed that electronic interaction should occur through the boron center. Moreover, intense emission was observed both in solution and solid. Finally, we demonstrated that the emission color of the PF_6_ salt was altered from crystal to amorphous by adding mechanical forces. Based on boron complexation and intrinsic solid-state luminescent characters, we achieved obtainment of emissive ionic materials with environmental responsivity.

## 1. Introduction

Most organic luminescent dyes show poor emission properties in solids due to aggregation-caused quenching (ACQ) induced mainly by non-specific intermolecular interactions. Therefore, for designing film-type sensors and devices based on organic materials, it is essential to load some mechanisms for suppressing ACQ. One of the promising strategies is to apply the class of molecules possessing AIE properties. AIE-active molecules can show intense emission only when they are aggregates. In the solution state, excited states are readily decayed by intramolecular interaction, while emission can be recovered in solid by suppressing molecular motions and intermolecular interactions. As a result, AIE behaviors can be realized. Furthermore, on the basis of potential environmental sensitivity of AIE-active molecules, various types of stimuli-responsive luminochromic materials and sensors have been developed by employing AIE-presenting skeletons [1,2,3]. For instance, by utilizing aggregation behaviors for signal amplification, a trace amount of water can be detected [4,5]. From this viewpoint, heteroatom-containing molecules with AIE properties are attractive candidates because potential environmental sensitivity of heteroatoms is available for expressing stimuli responsiveness [6,7,8,9]. We have also discovered AIE-active boron complexes and developed stimuli-responsive materials by using these molecules as an element-block [10,11,12,13], which is a building block containing heteroatoms for constructing functional materials [14,15,16,17]. For example, simply by connecting AIE-active boron complexes, AIE-active and solid-state luminescent polymers can be fabricated from heteroatom-containing molecules including boron complexes [18,19,20,21,22]. In particular, since stimuli responsiveness, such as luminochromism as well as intensity changes, was often observed, the series of luminescent sensors can be obtained [23,24,25,26,27,28,29]. By replacing boron with a different element, such as other group-13 elements, stimuli responsiveness and/or unique luminescent properties were observed [30,31,32,33]. From the changes in emission intensity and color, the target molecules or alteration of environmental factors can be monitored.

In conventional boron complexes, difluoride is very common because of its high stability and low synthetic difficulty. Meanwhile, some research groups tried replacing the two fluoride atoms with another unit [34,35,36,37,38,39,40,41,42]. The resulting spiro complexes have various unique features originating from cationic character and steric structures. For instance, boron complexes with two diketone ligands were synthesized [34,35]. These compounds showed interesting stimuli responsiveness, such as solvatochromism and thermochromism. Another paper reported that the central boron atom in the bis(1,9-oxido-phenalenyl)boron complex has an intriguing property [40,41,42]. In these research papers, boron complexes have the spiro structure, where each ligand perpendicularly bridges through the central boron atom. These non-planar spiro structure play key roles in organic electronic devices, such as OLEDs [43], organic phototransistors (OPTs) [44], and organic solid-state lasers (OSSLs) [45]. Spiro structures show chemical and thermal stability and good solubility because of their steric architectures. Further, their bulky structures play a role in lowering melting temperatures (*T*_m_s) and crystallinity. Therefore, spirobifluorene derivatives were used for OLEDs as amorphous emissive materials [45]. Therefore, we designed the new boron complexes consisting of the spiro structure to obtain solid-state luminescent properties and expected stimuli responsiveness in condensed states.

Herein, we report ionic salts consisting of the spiro structure with luminescent properties in solution and solid states. On the basis of the spiro structure with luminescent boron complexes, four types of ionic salts with variable counter anions were prepared. From the thermal analyses, it was observed that all salts have melting temperatures below 100 °C, indicating that the products can be classified as an ionic liquid according to the conventional definition. The ionic salts exhibit intense emission in solid as well as solution. In particular, the PF_6_ salt can form single crystal and show different luminescent colors between crystal and amorphous states. Finally, mechanochromic luminescence was observed from the PF_6_ salt. We can demonstrate the design of luminescent ionic materials based on boron coordination properties.

## 2. Results and Discussion

**Scheme 1:**
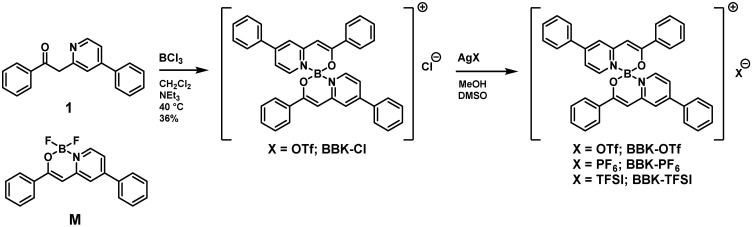
Syntheses of ionic salts.

Synthesis of the spirobi(boron ketoiminate) (BBK) structure and salt exchanges were performed according to Scheme 1 according to the previous study [20]. Boron trichloride was added to mixture solution of the ketoimine ligand **1** in the presence of triethylamine in CH_2_Cl_2_ and stirred at r.t. for 24 h. The salt BBK-Cl was obtained as a yellow solid after freeze-drying. We also prepared BKI as a model compound **M** [20]. From the characterization with ^1^H, ^13^C and ^11^B NMR spectroscopy and a mass measurement, we obtained the expected data. Further, by using BBK-Cl as a platform, we prepared the series of salt compounds with variable anion species. The DMSO solution of BBK-Cl was added to AgOTf in MeCN and stirred at r.t. for 3 h. After removal of the precipitated AgCl using filtration, the yellow solid was obtained through extraction followed by freeze-drying. With this protocol, three kinds of salts were obtained with trifluorosulfonate (OTf^−^), hexafluorophosphate (PF_6_^−^) and bis(trifluoromethanesulfonyl)imide (TFSI^−^) anions (Scheme 1). All products were characterized with NMR and mass spectrometry, and we concluded that the products have enough purity for further optical and thermal analyses.

**Figure 1:**
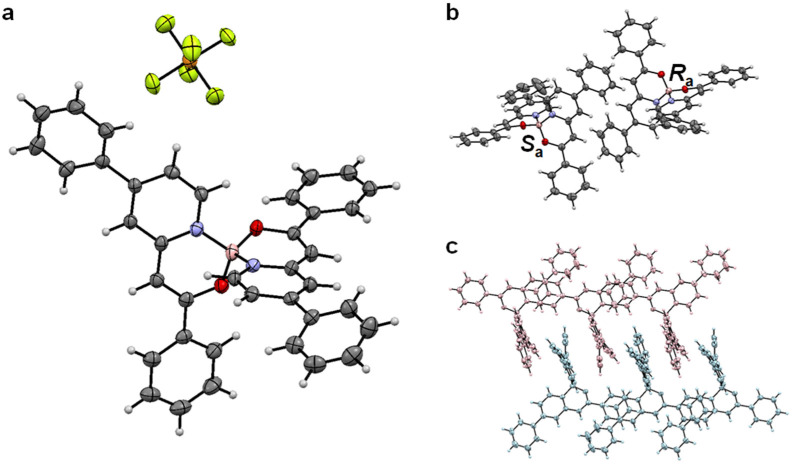
(**a**) ORTEP drawings of BBK-PF_6_ (black—C; blue—N; red—O; white—H; light green—F; orange—P). (**b**) Axial chirality pair of the BBK unit; eliminated PF_6_^−^. (**c**) Packing structure of chiral pair of the BBK unit; eliminated PF_6_^−^ (blue—*R*_a_, pink—*S*_a_).

Fortunately, it should be emphasized that BBK-PF_6_ formed a good single green crystal for analysis with X-ray crystallography. It was clearly shown that the compound has the expected structure and axial chirality (Figure 1). Accordingly, each *R*_a_ (light blue) and *S*_a_ (pink) formed dimer pairs and the layered structure, proposing that both π-conjugated planes should have an interaction. The distance between the closest planes was 3.46 Å, which is reasonable for the formation of π−π interaction. The structural data suggest that the symmetric property of PF_6_ anion and hydrogen bonds between fluoride and hydrogen could support crystallization.

Thermal properties, such as decomposition temperatures (*T*_d_s) and melting temperatures (*T*_m_s), were measured with thermogravimetric analysis (TGA) and differential scanning calorimetry (DSC), respectively (Table 1 and Figures S1 and S2). The *T*_d_ value of BBK-TFSI was higher than that of others. It is suggested that thermal motions should be suppressed in BBK-TFSI because the molecular weight of TFSI^−^ is the largest of the four anions. In addition, we estimated their weight losses from the TGA profiles and observed that each decrease was equal to molecular weight of anion species, meaning that initial degradation occurs at the anion moieties. In the DSC results, it was clearly shown that all compounds have *T*_m_ below 100 °C, indicating that all ionic salts can be classified as an ionic liquid [46,47,48]. Two ligands with the spiro structures could play a critical role in lowering *T*_m_s by disturbing intermolecular interaction in the crystalline state. Moreover, symmetric structures of cations might contribute to lowering *T*_m_s, as observed in the nano-cluster-containing ionic liquids [49,50].

**Figure 2:**
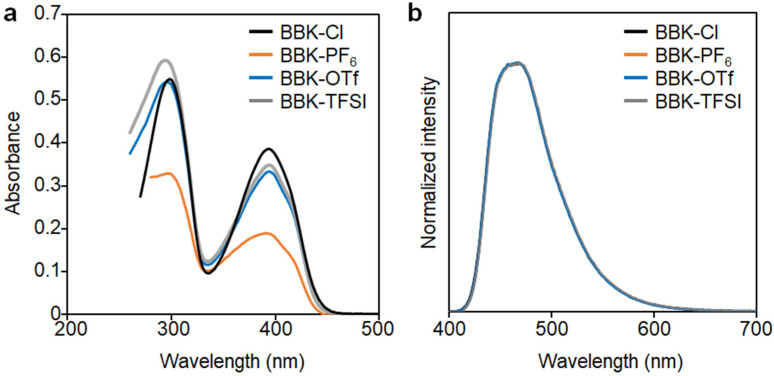
(**a**) UV−vis absorption and (**b**) emission spectra of the ionic salts in CH_2_Cl_2_ solution (1.0 × 10^−5^ mol/L). Emission spectra were obtained with the excitation light at *λ*_abs,max_.

To investigate electronic structures in the ground state, UV−vis absorption spectra in CH_2_Cl_2_ solution were measured. The peak wavelength of BBK-Cl was found at 392 nm (Figure 2a and Table 1). Compared with ketoiminate difluoride **M** as a model compound, the peak appeared in the longer-wavelength region. This result suggests that electronic interaction should be caused through the central boron atom. We also examined absorption properties through anion exchanges (Figure 2a and Table 1). Accordingly, significant changes were hardly observed, indicating anion species hardly affect the electronic structures of the ionic salts.

We measured emission spectra with three kinds of the ionic salts in dichloromethane (Figure 2b and Table 1). BBK-Cl showed the emission band in the blue region in the solution state, and slight differences were observed through anion exchange, indicating that anion hardly played a role in optical properties. Similar to the absorption properties, anion species hardly influence electronic structures of the ligand moieties in solution (Figure 2b and Table 1). Meanwhile, emission bands of the ionic salts were observed in the shorter wavelength region compared to that of **M**. According to the previous reports, the optical properties can be rationally explained [19,20]. The ligand potential shows a larger degree of structural relaxation in the excited state. Through the formation of the spiro structures, structural relaxation could be disturbed, followed by emission bands in the shorter-wavelength region. The smaller rate constant of non-radiation decay of the ionic salts comparing to that of **M** strongly supports the suppression of molecular motions in the excited state by the spiro formation.

**Figure 3:**
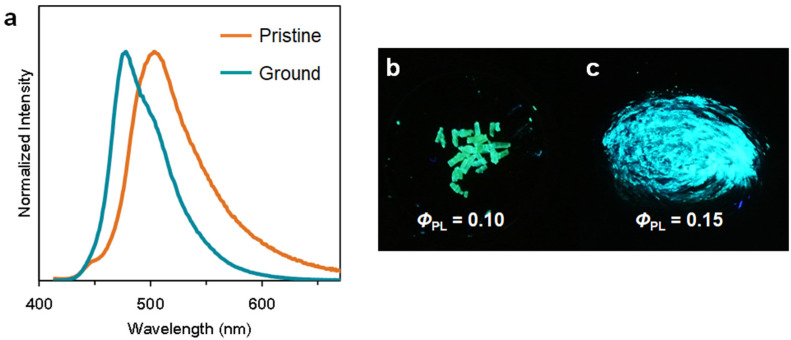
(**a**) Fluorescent spectra of BBK-PF_6_ in the solid states (orange—crystalline pristine sample; blue—ground sample). Photographs of BBK-PF_6_ under UV irradiation (**b**) before and (**c**) after grinding.

Since the crystalline sample was possible to obtain from BBK-PF_6_, we monitored changes in luminescent properties in solid (Figure 3 and Figure S4, Table 1 and Table S2). It should be emphasized that BBK-PF_6_ can exhibit emission in crystal (*Φ*_PL_ = 0.10, Table 1 and Table S1). The crystal sample of BBK-PF_6_ exhibited green emission with the peak at 507 nm (Figure 3a). When the sample was scratched, the emission band shifted to the blue region by 30 nm and emission color was changed to light blue (Figure 3b,c). These data represent that BBK-PF_6_ has a mechanochromic fluorescent property. This specific property should originate from changes in intermolecular interaction through π−π stacking during the morphology change from crystal to amorphous (Figure S3, Supplementary Materials) [51,52]. In the crystalline state, BBK-PF_6_ formed ordered structures and had strong π−π intermolecular interaction between each chirality pair (Figure 1c). As a result, the emission band was observed in the longer-wavelength region. On the other hands, when that ordered packing was crumbled by mechanical stress, intermolecular interaction should decrease. Consequently, the emission band appeared in the shorter wavelength region. Their quantum yields before and after grinding were 0.10 and 0.15, respectively. These data support that loss of π−π intermolecular interaction should be responsible for emission in the condensed state. It should be mentioned that the mechanochromic luminescent property was obtained only from BBK-PF_6_ which can form single crystals. Relatively higher crystallinity of the PF_6_ salt should be favorable for exhibiting luminochromism through molecular environmental changes.

## 3. Experimental Section

General: ^1^H (400 MHz), ^11^B (128 MHz), and ^13^C (100 MHz) NMR spectra were recorded on a JEOL JNM-EX400 or a JEOL JNM-AL400 spectrometers (JEOL ltd., Tokyo, Japan). In ^1^H and ^13^C NMR spectra, tetramethylsilane (TMS) was used as an internal standard in CDCl_3_, and ^11^B NMR spectra were referenced externally to BF_3_∙OEt_2_ (sealed capillary). UV−vis absorption spectra were recorded on a SHIMADZU UV-3600 spectrophotometer (Shimadzu Corporation, Kyoto, Japan). Photoluminescence (PL) spectra were measured with a HORIBA JOBIN YVON Fluorolog spectrofluorometer (HORIBA, Ltd., Kyoto, Japan), and photoluminescence quantum yields were calculated by the integrating sphere method. Fluorescence lifetime analyses were carried out on a Horiba FluoreCube spectrofluorometer system (HORIBA, Ltd., Kyoto, Japan); excitation at 375 nm using a UV diode laser (NanoLED-375L). Elemental analysis was performed at the Microanalytical Center of Kyoto University. DSC thermograms were carried out on a SII DSC 6220 instrument (Seiko Instruments Inc., Chiba, Japan). The sample on the aluminum pan was heated at the rate of 10 °C/min under nitrogen flowing (50 mL/min). Thermogravimetric analysis (TGA) was recorded on a Seiko Instruments Inc. (Chiba, Japan) EXSTAR TG/DTA6000. X-ray crystallographic analyses were carried out by Rigaku R-AXIS RAPID-F graphite-monochromated Mo K*α* radiation diffractometer with an imaging plate. A symmetry-related absorption correction was carried out using the program ABSCOR [53]. The analysis was carried out with direct methods (SHELX-97 [54] or SIR92 [55]) using Yadokari-XG [56]. The program ORTEP35 was used to generate the X-ray structural diagram [57,58]. Powder X-ray diffraction (PXRD) patterns were taken by using CuKα radiation with Rigaku Miniflex (Rigaku Corporation, Tokyo, Japan).

Synthesis of **M**: BF_3_⋅Et_2_O (6.2 mL, 7.11 g, 50.1 mmol) was added to the solution of **1** [20] (1.37 g, 5.0 mmol) in the mixed solvent of CH_2_Cl_2_ (30 mL) and NEt_3_ (6 mL). The reaction mixture was refluxed under Ar atmosphere for 24 h and then cooled at r.t. The organic layer was washed with water (100 mL × 2) and brine (100 mL), dried over anhydrous magnesium sulfate, and concentrated by a rotary evaporator. The resulting solid was purified by silica gel column chromatography eluted with hexane/AcOEt (2/1). The product **M** was obtained after it was recrystallized from ethanol as an orange crystal (0.81 g, 50%). ^1^H NMR (CDCl_3_): δ 8.47 (1H, d, *J* = 6.2 Hz, Ar-H), 7.97 (2H, dd, *J* = 6.8, 1.7 Hz), 7.70 (2H, dd, *J* = 5.9, 2.1 Hz), 7.56 (1H, s), 7.55 (2H, d, *J* = 2.6 Hz), 7.49 (2H, d, *J* = 5.3 Hz), 7.45 (3H, m), 6.44 (1H, s) ppm. ^13^C NMR (CDCl_3_): δ 163.1, 153.7, 151.9, 140.2, 136.1, 134.3, 130.9, 130.8, 129.5, 128.5, 127.3, 126.6, 119.5, 118.5, 93.4 ppm. ^11^B NMR (CDCl_3_): δ 1.47 ppm. HRMS (ESI): Calculated for [M + H]^+^, 322.1209; found, *m*/*z* 322.1209.

Synthesis of **BBK-Cl**: BCl_3_ in CH_2_Cl_2_ solution (1.6 mL, 187 mg, 1.60 mmol) was added to the solution of **1** (918 mg, 3.36 mmol) in the mixed solvent of CH_2_Cl_2_ (24 mL) and NEt_3_ (6.4 mL). The reaction mixture was stirred at r.t. under Ar atmosphere for 12 h. After removing solvents using a rotary evaporator, the yellow residue was dissolved into DMSO and precipitated into a large amount of Et_2_O. The precipitation was recrystallized from Et_2_O and MeOH. The product BBK-Cl was obtained as a yellow solid (326 mg, 36%). ^1^H NMR (DMSO-*d*_6_): δ 8.44 (1H, d, *J* = 6.6 Hz, Ar-H), 8.34 (1H, d, *J* = 2.0 Hz, Ar-H), 8.01 (2H, dd, *J* = 5.1, 1.7 Hz, Ar-H), 7.94–7.90 (3H, m, Ar-H), 7.70–7.50 (6H, m, Ar-H), 7.33 (1H, s, Ar-H) ppm. ^13^C NMR (DMSO-*d*_6_): δ 153.7, 151.0, 141.8, 134.6, 132.8, 131.5, 131.4, 129.6, 129.0, 127.6, 127.1, 126.0, 120.8, 120.3, 95.0 ppm. ^11^B NMR (DMSO-*d*_6_): δ 4.12 ppm. HRMS (ESI): Calculated for [M + H]^+^, 555.2238; found, *m*/*z* 555.2235.

Synthesis of **BBK-PF_6_**: BBK-Cl (200 mg, 0.34 mmol) was dissolved into MeOH (5 mL) and added to the solution of silver hexafluorophosphate (105 mg, 0.37 mmol) in acetonitrile (5 mL). After stirring at r. t. for 2 h, the solution was filtered to remove white precipitate. The obtained precipitate was extracted with CH_2_Cl_2_ and washed with water twice. The resulting yellow solution was concentrated using a rotary evaporator and redissolved into benzene. After freeze-drying, BBK-PF_6_ was obtained as a yellow solid (80%, 190 mg). ^1^H NMR (CDCl_3_): δ 8.08 (1H, d, *J* = 6.6 Hz, Ar-H), 7.92 (2H, dd, *J* = 7.8, 1.2 Hz, Ar-H), 7.80–7.76 (3H, m, Ar-H), 7.94–7.90 (1H, dd, *J* = 6.6, 2.0 Hz, Ar-H), 7.60–7.44 (6H, m, Ar-H), 6.81 (1H, s, Ar-H) ppm. ^13^C NMR (DMSO-*d*_6_): δ 153.8, 151.0, 141.8, 134.6, 132.8, 131.5, 131.4, 129.6, 129.0, 127.6, 127.1, 126.0, 120.8, 120.3, 95.1 ppm. ^11^B NMR (DMSO-*d*_6_): δ 4.12 ppm. HRMS (ESI): Calculated for [M + H]^+^, 555.2238; found, *m*/*z* 555.2235. Calculated for [PF_6_]^−^, 144.9647; found, *m*/*z* 144.9644. CCDC #: 2071316.

Synthesis of **BBK-OTf**: BBK-OTf was prepared from BBK-Cl (200 mg, 0.34 mmol) and silver trifluoromethanesulfonate (96 mg, 0.37 mmol) as yellow solid according to the same method with BBK-PF_6_. ^1^H NMR (DMSO-*d*_6_): δ 8.44 (1H, d, *J* = 6.6 Hz, Ar-H), 8.32 (1H, s, Ar-H), 8.01 (2H, dd, *J* = 5.1, 1.7 Hz, Ar-H), 7.94–7.90 (3H, m, Ar-H), 7.70–7.50 (6H, m, Ar-H), 7.31 (1H, s, Ar-H) ppm. ^13^C NMR (DMSO-*d*_6_): δ 153.7, 151.0, 144.0, 141.8, 134.6, 132.8, 131.5, 131.4, 129.6, 129.0, 127.7, 127.2, 126.0, 120.8, 120.3, 95.1 ppm. ^11^B NMR (DMSO-*d*_6_): δ 4.12 ppm. HRMS (ESI): Calculated for [M + H]^+^, 555.2238; found, m/z 555.2235. HRMS (ESI): Calculated for [M + H]^+^, 555.2238; found, *m*/*z* 555.2236. Calculated for [OTf]^−^, 148.9526; found, *m*/*z* 148.9522.

Synthesis of **BBK-TFSI**: BBK-TFSI was prepared from BBK-Cl (200 mg, 0.34 mmol) and silver bis(trifluoromethanesulfonyl)imide (144 mg, 0.37 mmol) as yellow solid according to the same method with BBK-PF_6_. ^1^H NMR (DMSO-*d*_6_): δ 8.44 (1H, d, *J* = 6.6 Hz, Ar-H), 8.32 (1H, d, *J* = 2.0 Hz, Ar-H), 8.01 (2H, dd, *J* = 7.3, 3.6 Hz, Ar-H), 7.94–7.90 (3H, m, Ar-H), 7.70–7.50 (6H, m, Ar-H), 7.31 (1H, s, Ar-H) ppm. ^13^C NMR (DMSO-*d*_6_): δ 158.6, 153.8, 141.8, 140.2, 134.6, 132.8, 131.5, 131.4, 129.6, 129.0, 127.6, 127.1, 126.0, 120.8, 120.3, 95.1 ppm. ^11^B NMR (DMSO-*d*_6_): δ 4.12 ppm. HRMS (ESI): Calculated for [M + H]^+^, 555.2238; found, *m*/*z* 555.2235. HRMS (ESI): Calculated for [M + H]^+^, 555.2238; found, *m*/*z* 555.2236. Calculated for [TFSI]^−^, 279.9178; found, *m*/*z* 279.9175.

## 4. Conclusions

By employing the luminescent boron complex structure, we obtained luminescent ionic salts with various kinds of anions. Since the *T*_m_ values were found below 100 °C, these salts can be classified as an ionic liquid. All molecules show intense emission not only in solution but also in solid. By replacing the counter anion, the crystallinity can be altered. In particular, it was found that the PF_6_ salt can form a single crystal and an X-ray analysis was applicable. Furthermore, we accomplished detection of luminochromism from crystal to amorphous state using mechanical stimuli. It is proposed that our stimuli-responsive luminescent materials might be potentially applicable to introduce environment-monitoring ability in the conventional usages of ionic liquids, such as electrolytes in lithium batteries and thermal-resistant reaction solvents.

## Figures and Tables

**Table 1:** Optical properties of the ionic salts with variable anion.

	BBK-Cl	BBK-PF_6_	BBK-OTf	BBK-TFSI	M *^f^*
*λ*_abs,sol_ [nm] *^a^*	391	391	393	394	380
*ε* [M^−1^cm^−1^] *^a^*	39,200	19,000	33,300	34,800	22,300
*λ*_em,solution_ [nm] *^a^*	453	453	455	453	460
*Φ*_solution_ *^a,b^*	0.26	0.24	0.26	0.26	0.35
*τ* [ns] *^c^*	1.3	1.4	1.4	1.4	0.5
	(*χ*^2^ = 1.12)	(*χ*^2^ = 1.15)	(*χ*^2^ = 1.03)	(*χ*^2^ = 1.15)	(*χ*^2^ = 1.01)
*k*_f_ [× 10^8^ s^−1^]	1.8	1.7	1.8	1.8	3.7
*k*_nr_ [× 10^8^ s^−1^]	5.7	5.4	5.2	5.3	18.1
*T*_d_ (°C) *^d^*	214	218	193	233	
*T*_m_ (°C) *^e^*	75	65	68	55	

*^a^* Measured in CH_2_Cl_2_ (1.0 × 10^−5^ mol/L). *^b^* Calculated as an absolute value determined using an integrated sphere method. *^c^* Fluorescence lifetime: excited at 375 nm, detected at *λ*_em,sol_. *^d^* Determined from the onset in TGA profiles. *^e^* Determined from DSC. *^f^* Reprinted with permission from ref. [20]. Copyright 2017 John Wiley and Sons.

## Data Availability

The data presented in this study are available in article and Supplementary Materials.

## Supplementary Materials

The following supporting information can be downloaded at: https://www.mdpi.com/article/10.3390/molecules27113438/s1, Figure S1: TGA diagrams of BBK salts under N_2_ flowing; Figure S2: DSC profiles of BBK salts; Figure S3: XRD profiles of BBK-PF6 before and after the mechanical treatment; Figure S4: Solid-state luminescent properties of BBK salts; Figure S5: ^1^H NMR spectrum of BBK-Cl in DMSO-*d*_6_; Figure S6: ^13^C NMR spectrum of BBK-Cl in DMSO-*d*_6_; Figure S7: ^11^B NMR spectrum of BBK-Cl in DMSO-*d*_6_; Figure S8: ^1^H NMR spectrum of BBK-PF_6_ in CDCl_3_; Figure S9: ^13^C NMR spectrum of BBK-PF_6_ in DMSO-*d*_6_; Figure S10: ^11^B NMR spectrum of BBK-PF_6_ in DMSO-*d*_6_; Figure S11: ^1^H NMR spectrum of BBK-OTf in DMSO-*d*_6_; Figure S12: ^13^C NMR spectrum of BBK-OTf in DMSO-*d*_6_; Figure S13: ^11^B NMR spectrum of BBK-OTf in DMSO-*d*_6_; Figure S14: ^1^H NMR spectrum of BBK-TFSI in DMSO-*d*_6_; Figure S15: ^13^C NMR spectrum of BBK-TFSI in DMSO-*d*_6_; Figure S16: ^11^B NMR spectrum of BBK-TFSI in DMSO-*d*_6_; Table S1: Optical properties of BBK-PF_6_; Table S2: Solid-state luminescent properties of BBK salts.

## Sample Availability

Samples of the compounds are not available from the authors.
