# Supplementary material for: Modulation of Properties by Ion Changing Based on Luminescent Ionic Salts Consisting of Spirobi(boron ketoiminate)
Source: Molecules. 2022 May 26;27(11):3438. doi: 10.3390/molecules27113438 (PMC9182478; doi:10.3390/molecules27113438)
Supplement: Supplementary file 1 [file molecules-27-03438-s001.zip › molecules-1738255-supplementary-proof done.pdf]

Supporting Information

Modulation of Properties by Ion Changing Based on Luminescent Ionic Salts Consisting of Spirobi(boron ketoiminate)

Kazumasa Suenaga, Shunichiro Ito, Kazuo Tanaka* and Yoshiki Chujo

Department of Polymer Chemistry, Graduate School of Engineering, Kyoto University

Katsura, Nishikyo-ku, Kyoto 615-8510, Japan

Tel: +81-75-383-2604

Fax: +81-75-383-2605

E-mail: tanaka@poly.synchem.kyoto-u.ac.jp

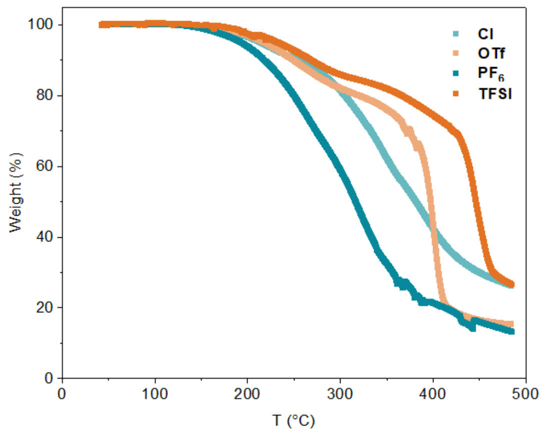

Figure S1. TGA diagrams of BBK salts under N_2 flowing.

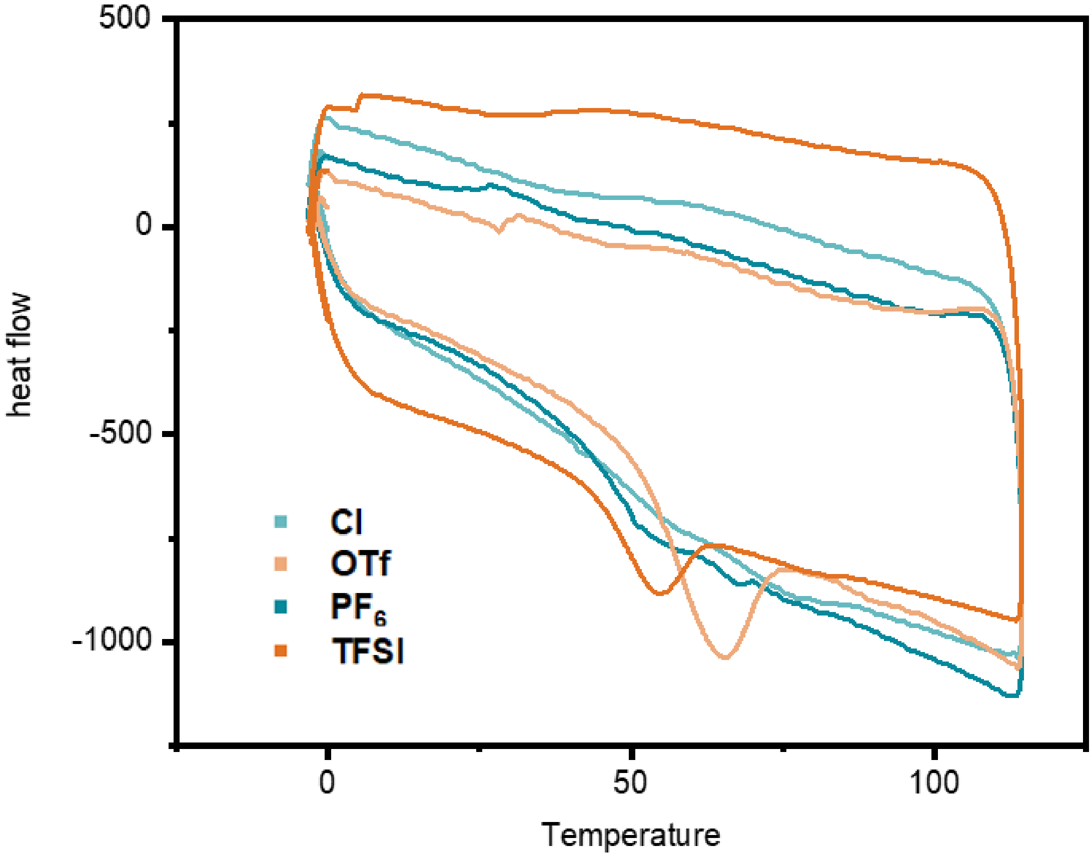

Figure S2. DSC profiles of BBK salts.

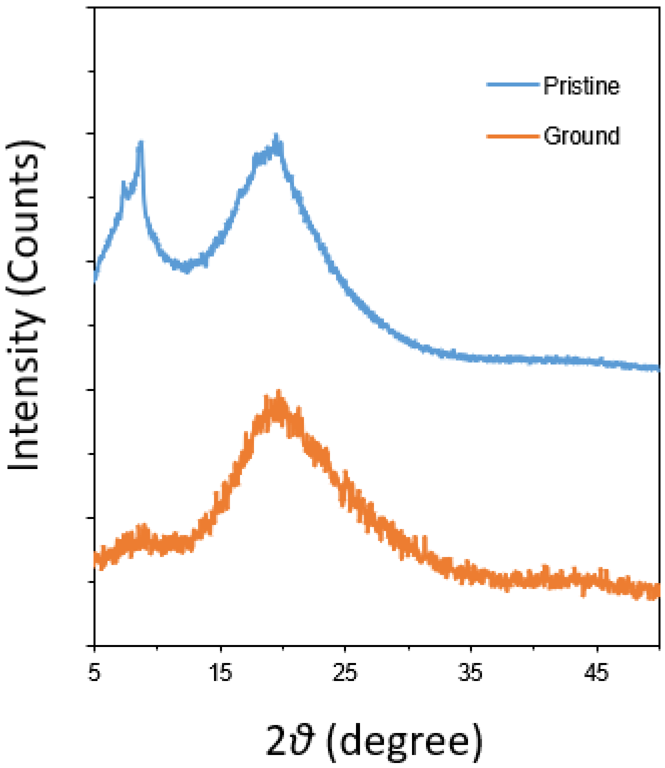

Figure S3. XRD profiles of BBK-PF_6 before and after the mechanical treatment.

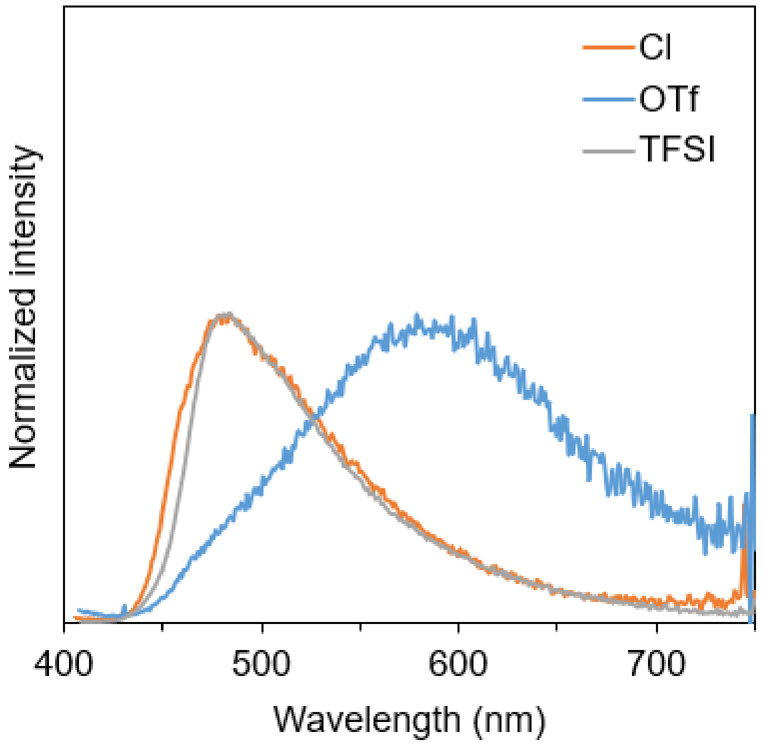

Figure S4. Solid-state luminescent properties of BBK salts.

Table S1. Optical properties of BBK-PF_6

	λ_{ab} [nm]	λ_{em} [nm]	Φ
Solution	391	453	0.26
Crystal	-	506	0.10
Amorphous	-	477	0.15

Table S2. Solid-state luminescent properties of BBK salts

salts	Cl	OTf	TFSI
λ_{em} (nm)	484	579	484
Φ_{em}	0.026	0.015	0.063

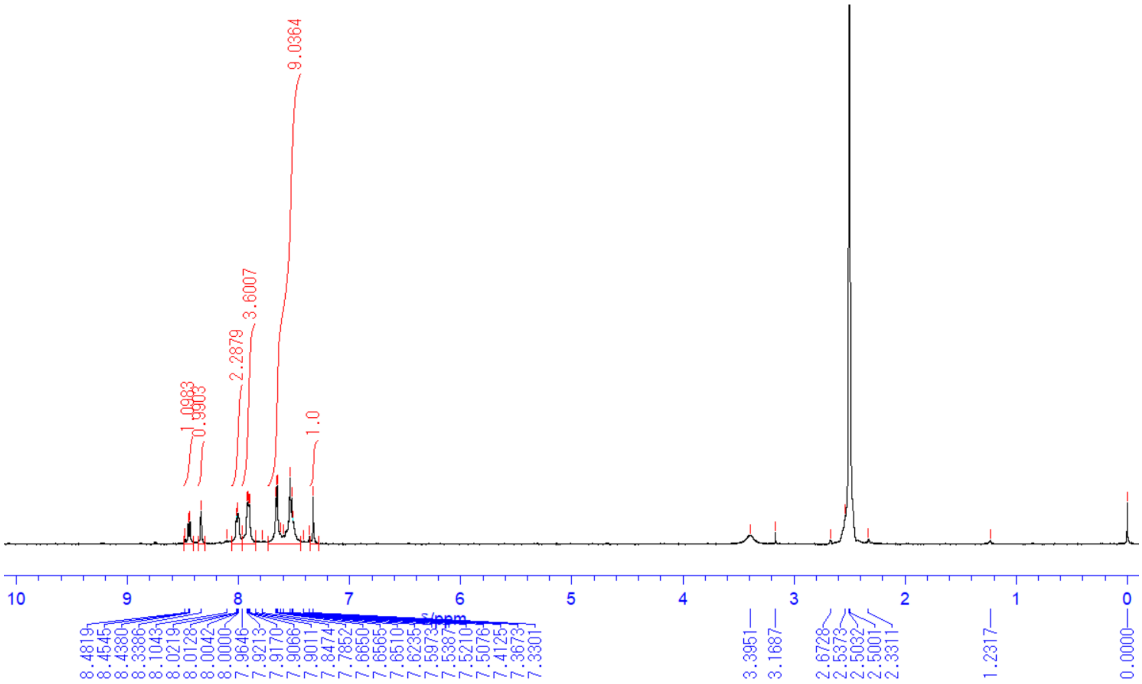

Figure S5. ^{1}H NMR spectrum of BBK-Cl in DMSO-d_6.

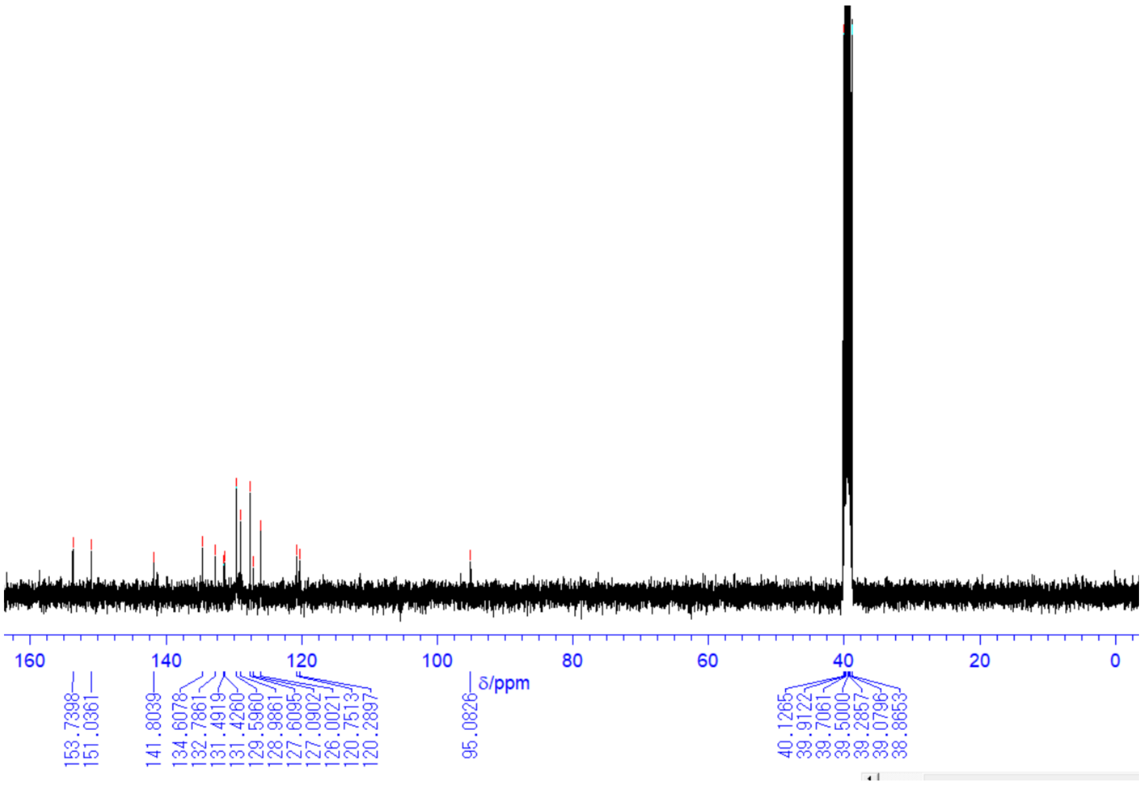

Figure S6. ^{13}C NMR spectrum of BBK-Cl in DMSO-d_6.

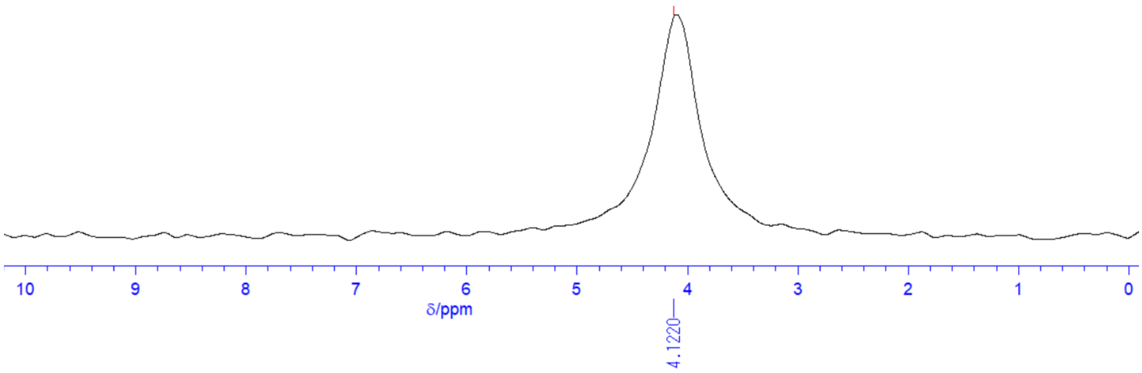

Figure S7. ^{11}B NMR spectrum of BBK-Cl in DMSO-d_6.

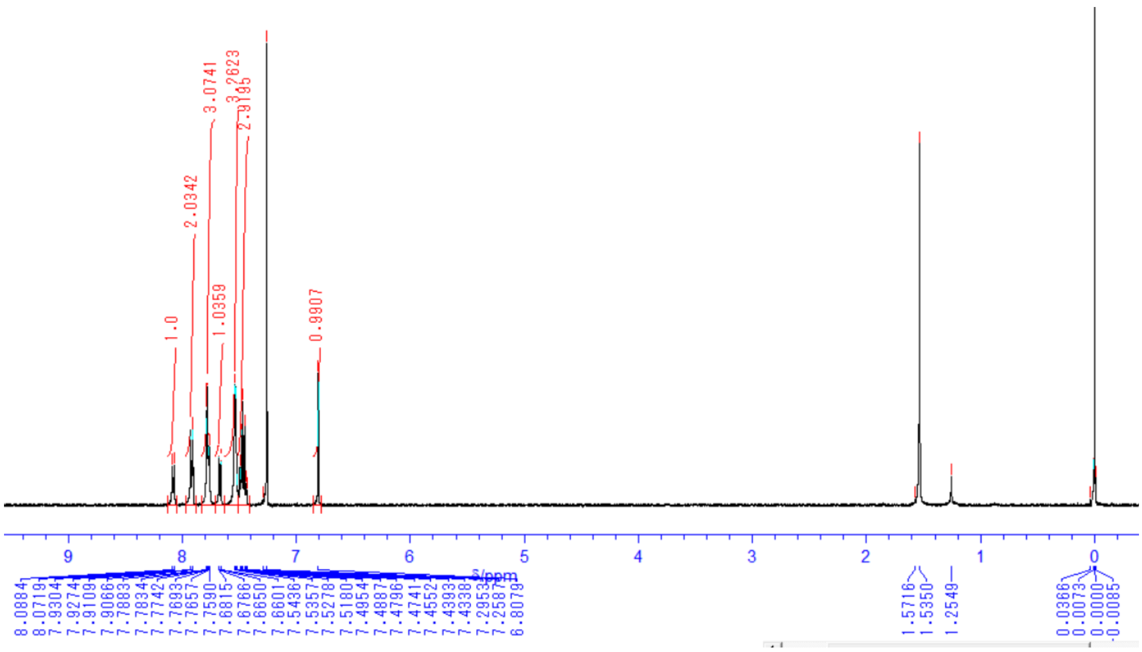

Figure S8. ^{1}H NMR spectrum of BBK-PF_6 in $CDCl_3$.

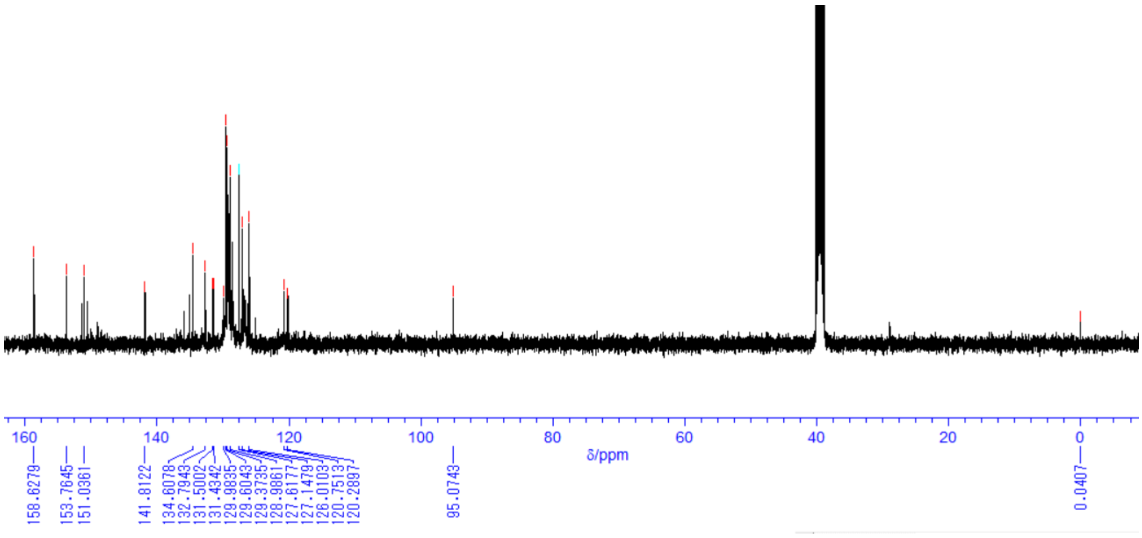

Figure S9. ^{13}C NMR spectrum of BBK-PF_6 in DMSO-d_6.

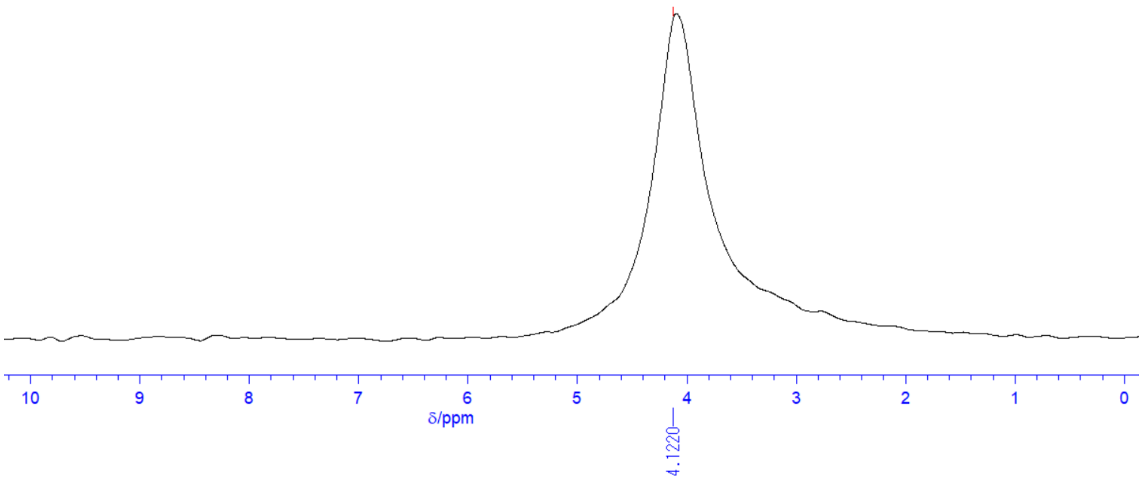

Figure S10. ^{11}B NMR spectrum of BBK-PF_6 in DMSO-d_6.

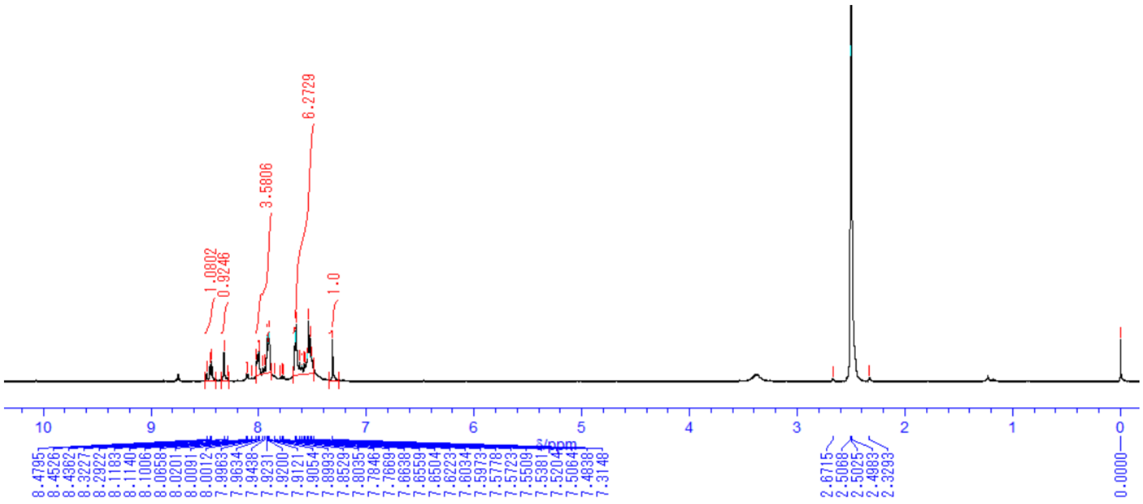

Figure S11. ^{1}H NMR spectrum of BBK-OTf in DMSO-d_6.

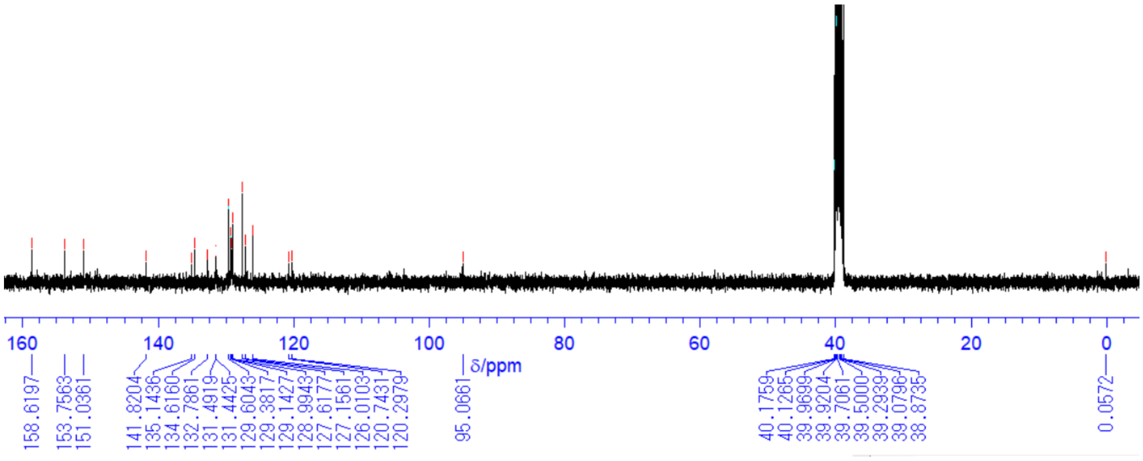

Figure S12. ^{13}C NMR spectrum of BBK-OTf in DMSO-d_6.

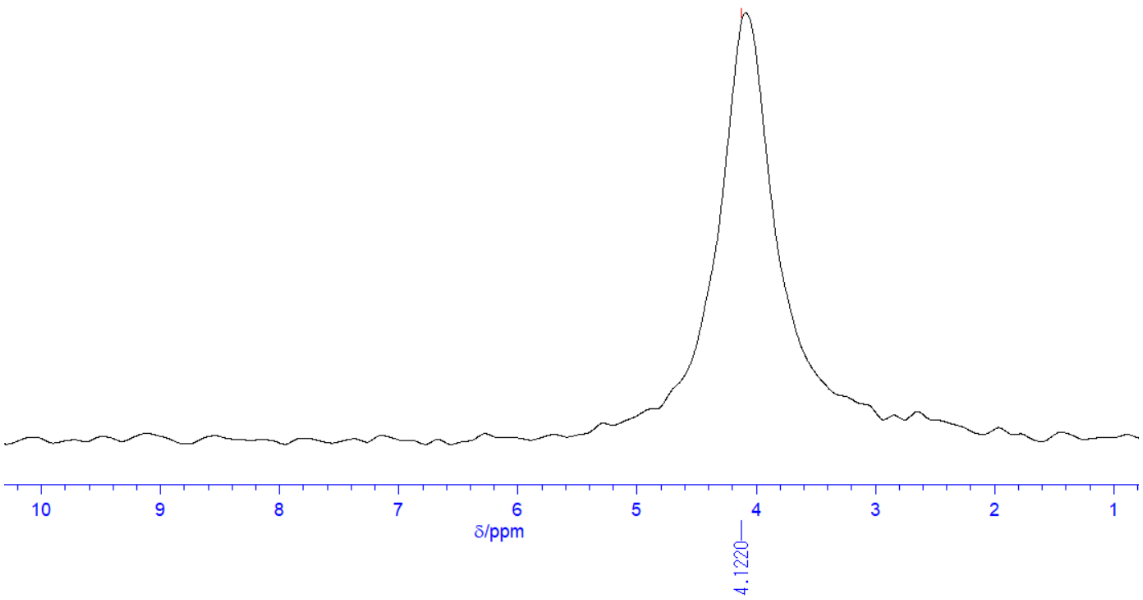

Figure S13. ^{11}B NMR spectrum of BBK-OTf in DMSO-d_6.

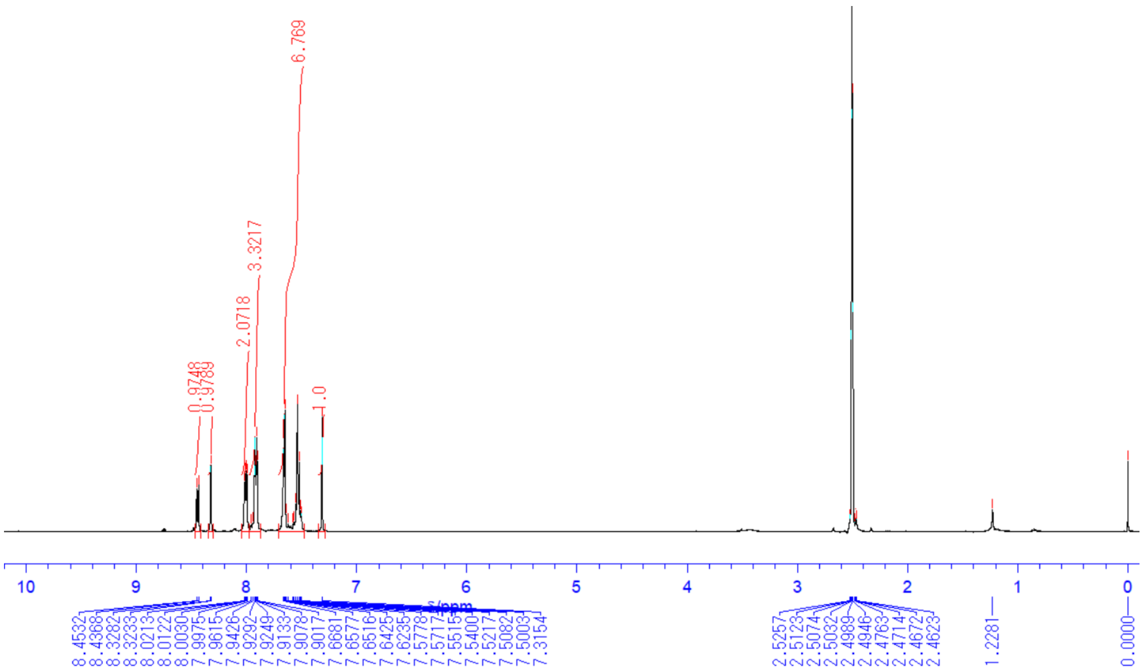

Figure S14. ^{1}H NMR spectrum of BBK-TFSI in DMSO-d_6.

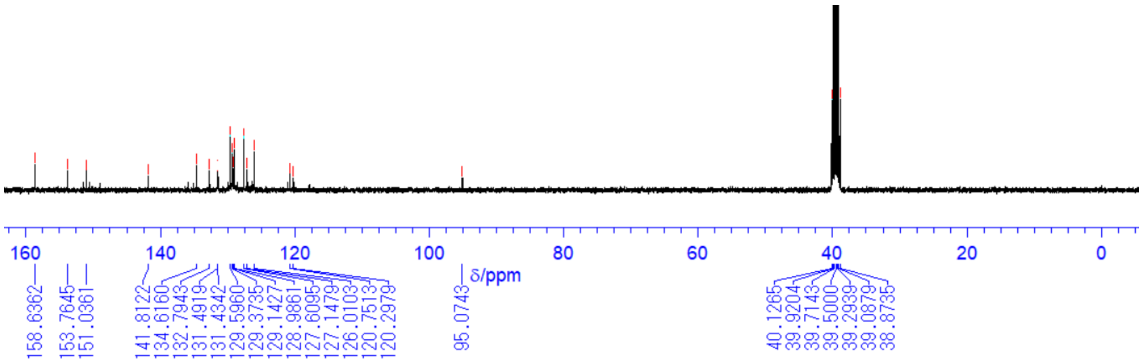

Figure S15. ^{13}C NMR spectrum of BBK-TFSI in DMSO-d_6.

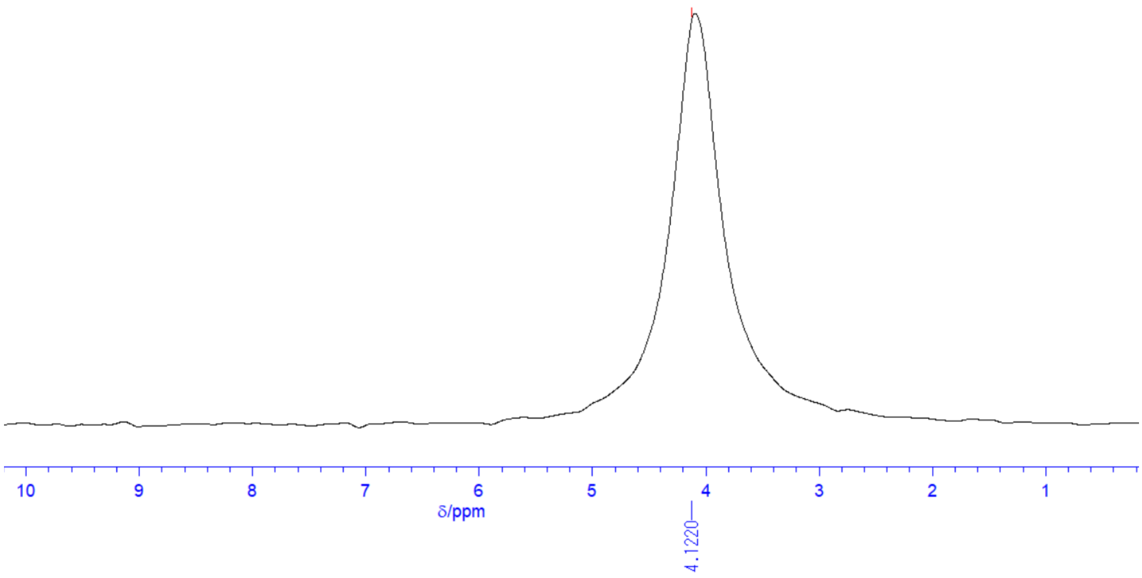

Figure S16. ^{11}B NMR spectrum of BBK-TFSI in DMSO-d_6.
