# Supplementary material for: Modulation of Properties by Ion Changing Based on Luminescent Ionic Salts Consisting of Spirobi(boron ketoiminate)
Source: Molecules. 2022 May 26;27(11):3438. doi: 10.3390/molecules27113438 (PMC9182478; doi:10.3390/molecules27113438)

checkCIF/PLATON report

You have not supplied any structure factors. As a result the full set of tests cannot be run.

THIS REPORT IS FOR GUIDANCE ONLY. IF USED AS PART OF A REVIEW PROCEDURE FOR PUBLICATION, IT SHOULD NOT REPLACE THE EXPERTISE OF AN EXPERIENCED CRYSTALLOGRAPHIC REFEREE.

No syntax errors found.   CIF dictionary   Interpreting this report

Datablock: SFBKI

Bond precision:	C-C = 0.0042 A		Wavelength=0.71075
Cell:	a=16.7414(10)	b=13.6507(10)	c=28.1368(17)
	alpha=90	beta=90.054(6)	gamma=90
Temperature:	93 K		

	Calculated	Reported
Volume	6430.2(7)	6430.2(7)
Space group	P 21/n	P 21/n
Hall group	-P 2yn	-P 2yn
Moiety formula	C38 H28 B N2 O2, F6 P	C38 H28 B F6 N2 O2 P
Sum formula	C38 H28 B F6 N2 O2 P	C38 H28 B F6 N2 O2 P
Mr	700.40	700.40
Dx,g cm-3	1.447	1.447
Z	8	8
Mu (mm-1)	0.160	0.160
F000	2880.0	2880.0
F000'	2882.52	
h,k,lmax	21,17,36	21,17,36
Nref	14745	14550
Tmin,Tmax	0.923,0.953	0.489,1.000
Tmin'	0.923	

Correction method= # Reported T Limits: Tmin=0.489 Tmax=1.000
AbsCorr = MULTI-SCAN

Data completeness= 0.987   Theta(max)= 27.485

R(reflections)= 0.0766(9535)   wR2(reflections)= 0.1965(14550)

S = 1.080   Npar= 901

The following ALERTS were generated. Each ALERT has the format
      test-name_ALERT_alert-type_alert-level.
Click on the hyperlinks for more details of the test.

Alert level C

```
RINTA01_ALERT_3_C  The value of Rint is greater than 0.12
            Rint given   0.132
PLAT020_ALERT_3_C The Value of Rint is Greater Than 0.12 .........      0.132 Report
PLAT085_ALERT_2_C SHELXL Default Weighting Scheme is not Optimized     Please Check
PLAT220_ALERT_2_C NonSolvent   Resd 2  C   Ueq(max)/Ueq(min) Range        3.6 Ratio
PLAT241_ALERT_2_C High   'MainMol' Ueq as Compared to Neighbors of       C02S Check
PLAT340_ALERT_3_C Low Bond Precision on  C-C Bonds ...............     0.0042 Ang.
PLAT410_ALERT_2_C Short Intra H...H Contact  H14      ..H22      .       1.99 Ang.
                                                      x,y,z  =      1_555 Check
PLAT410_ALERT_2_C Short Intra H...H Contact  H18      ..H46      .       1.99 Ang.
                                                      x,y,z  =      1_555 Check
```

Alert level G

```
PLAT012_ALERT_1_G N.O.K.   _shelx_res_checksum Found in CIF ......     Please Check
PLAT042_ALERT_1_G Calc. and Reported Moiety Formula Strings Differ     Please Check
PLAT244_ALERT_4_G Low    'Solvent' Ueq as Compared to Neighbors of       P001 Check
PLAT244_ALERT_4_G Low    'Solvent' Ueq as Compared to Neighbors of       P002 Check
PLAT720_ALERT_4_G Number of Unusual/Non-Standard Labels ..........        100 Note
PLAT790_ALERT_4_G Centre of Gravity not Within Unit Cell: Resd.  #          4 Note
              F6 P
PLAT802_ALERT_4_G CIF Input Record(s) with more than 80 Characters          1 Info
PLAT883_ALERT_1_G No Info/Value for _atom_sites_solution_primary .     Please Do !
PLAT965_ALERT_2_G The SHELXL WEIGHT Optimisation has not Converged     Please Check
PLAT967_ALERT_5_G Note: Two-Theta Cutoff Value in Embedded .res ..       55.0 Degree
```

   0 **ALERT level A** = Most likely a serious problem - resolve or explain
   0 **ALERT level B** = A potentially serious problem, consider carefully
   8 **ALERT level C** = Check. Ensure it is not caused by an omission or oversight
  10 **ALERT level G** = General information/check it is not something unexpected

   3 ALERT type 1 CIF construction/syntax error, inconsistent or missing data
   6 ALERT type 2 Indicator that the structure model may be wrong or deficient
   3 ALERT type 3 Indicator that the structure quality may be low
   5 ALERT type 4 Improvement, methodology, query or suggestion
   1 ALERT type 5 Informative message, check

It is advisable to attempt to resolve as many as possible of the alerts in all categories. Often the minor alerts point to easily fixed oversights, errors and omissions in your CIF or refinement strategy, so attention to these fine details can be worthwhile. In order to resolve some of the more serious problems it may be necessary to carry out additional measurements or structure refinements. However, the purpose of your study may justify the reported deviations and the more serious of these should normally be commented upon in the discussion or experimental section of a paper or in the "special_details" fields of the CIF. checkCIF was carefully designed to identify outliers and unusual parameters, but every test has its limitations and alerts that are not important in a particular case may appear. Conversely, the absence of alerts does not guarantee there are no aspects of the results needing attention. It is up to the individual to critically assess their own results and, if necessary, seek expert advice.

Publication of your CIF in IUCr journals

A basic structural check has been run on your CIF. These basic checks will be run on all CIFs submitted for publication in IUCr journals (*Acta Crystallographica*, *Journal of Applied Crystallography*, *Journal of Synchrotron Radiation*); however, if you intend to submit to *Acta Crystallographica Section C* or *E* or *IUCrData*, you should make sure that full publication checks are run on the final version of your CIF prior to submission.

Publication of your CIF in other journals

Please refer to the *Notes for Authors* of the relevant journal for any special instructions relating to CIF submission.

PLATON version of 19/02/2022; check.def file version of 19/02/2022

Datablock SFBKI - ellipsoid plot

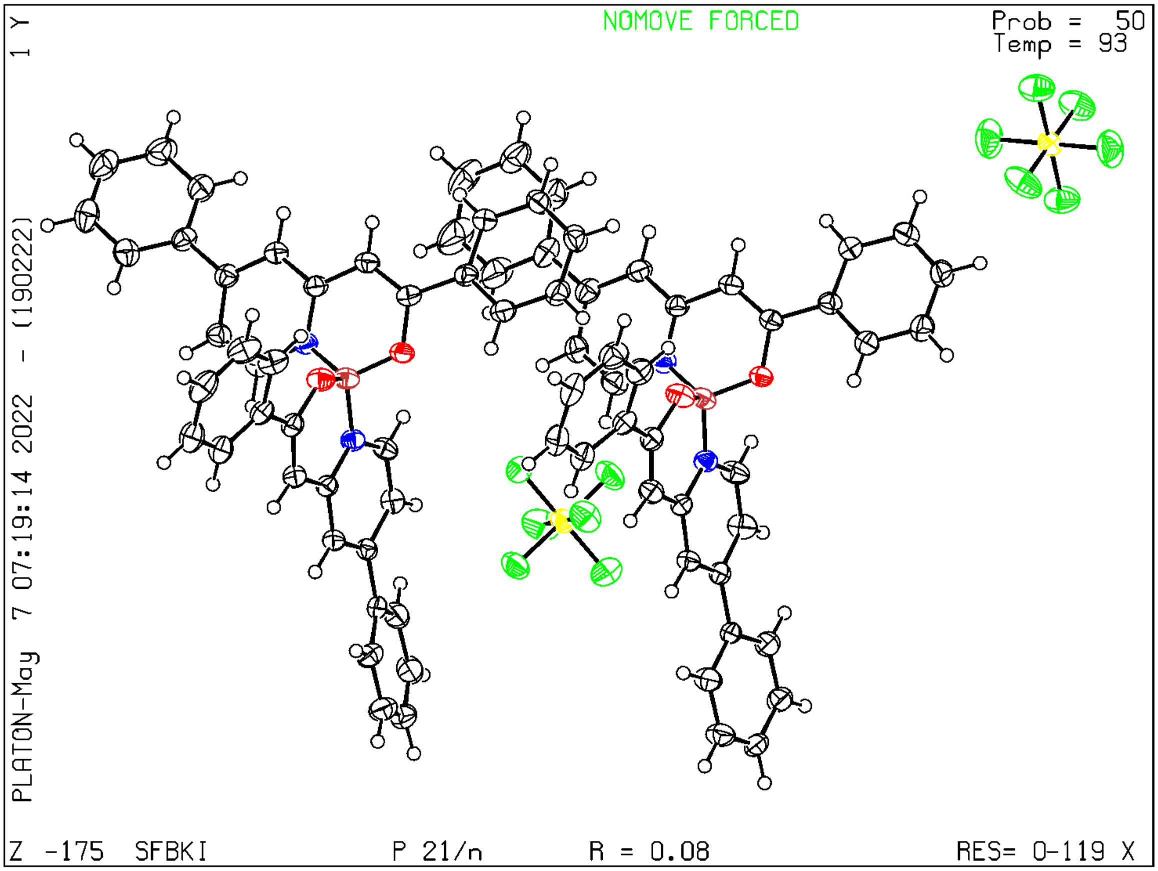

Supplement: Supplementary file 1 [file molecules-27-03438-s001.zip › SFBKI(H)-PF6_ito_checkcif.pdf]
